# Characteristics of electrospun chitosan/carbon nanotube coatings deposited on AZ31 magnesium alloy

**DOI:** 10.1007/s10856-022-06703-1

**Published:** 2023-01-11

**Authors:** Shaghayegh Vahedi, Rouhollah Mehdinavaz Aghdam, Mahmoud Heydarzadeh Sohi, Ali Hossein Rezayan

**Affiliations:** 1 School of Metallurgy and Materials Engineering, College of Engineering, University of Tehran, P.O. Box: 11155-4563, Tehran, Iran; 2 Division of Nanobiotechnology, Department of Life Science Engineering, Faculty of New Sciences & Technologies, University of Tehran, P.O. Box: 11155-4563, Tehran, Iran

**Keywords:** Magnesium, Nanocomposite coating, Chitosan, Carbon nanotubes, Nanofiber

## Abstract

Mg-based biomaterials are commonly used as biodegradable orthopedic implants (e.g., bone regeneration applications). However, achieving high biocompatibility and corrosion resistance has remained a challenge to be tackled. In this work, to investigate various fabricated coatings (with and without pre- anodizing), five categories of samples are considered: (a) bare Mg alloy (Mg), (b) Anodized Mg alloy (Mg-A), (c) CS-coated Mg alloy (Mg-C), (d) CS-coated anodized Mg alloy (Mg-AC), and (e) CS-CNT-coated anodized Mg alloy (Mg-ACC). These samples were characterized by using Field Emission Scanning Electron Microscopes (FE-SEM), Energy Dispersive Spectroscopy (EDS), Fourier Transform Infrared Spectroscopy (FT-IR), and Raman Spectroscopy. The adhesion within the coated samples was compared. Then, the effects of the coatings were evaluated by comparing wettability, corrosion behavior, and biocompatibility for bare and coated samples. The adhesion test showed that the coatings exhibited higher adhesion for Mg-AC and Mg-ACC compared to Mg-C. Desired wettability was achieved as the contact angles of coated samples were in the range of 55°– 65°. Electrochemical impedance and polarization as well as immersion tests showed higher corrosion resistance for coated samples. The composite coated sample showed improved cell adhesion since the osteoblast cells covered almost the entire surface of the sample. Moreover, osteoblast cell viability for the sample was around 40% higher than that of the bare sample.

**Graphical abstract:**

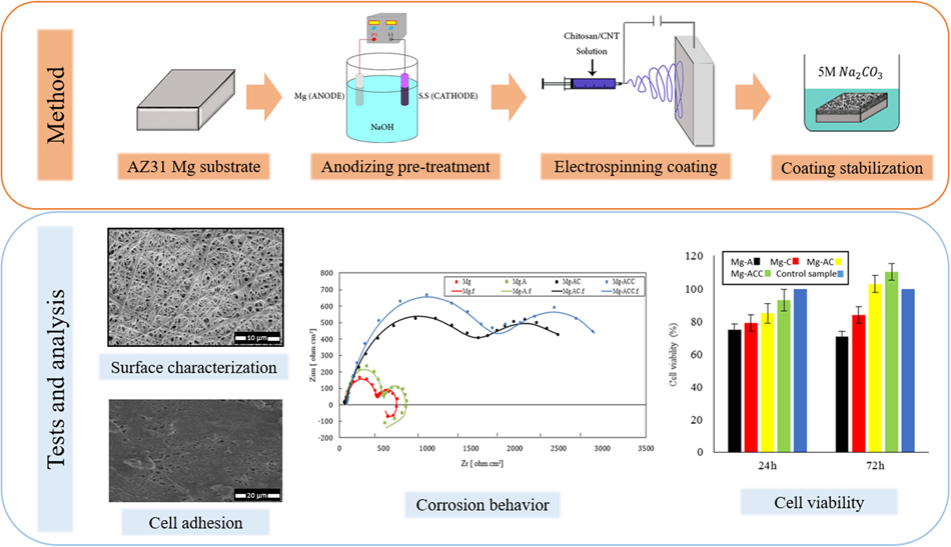

## Introduction

Magnesium (Mg) and its alloys are highly regarded for use in biodegradable orthopedic implants [1]. Unlike conventional metallic implants, magnesium implants can be resorbed by the body [2]. They react with body fluids and get removed through the body’s metabolism without producing any toxic by-product. Therefore, there is no need for a secondary surgery to remove the implants. In addition, Mg has excellent mechanical properties, including low density and a high strength to weight ratio. Also, their modulus of elasticity (*E* = 41–45 GPa) is very close to that of human bone which results in reduced stress shielding and failure of implants [3].

One disadvantage of Mg alloys is that they corrode rapidly in body fluids resulting in a high rate of hydrogen gas (H_2_) release [4]. This causes degraded mechanical integrity before new bone regeneration. High corrosion rates also cause the formation of harmful H_2_ gas pockets which can cause necrosis [5]. Furthermore, the hydroxide layer formed on the Mg substrate in the body fluid cannot protect it against corrosion [6]. These issues limit the use of Mg alloys for bone implant applications [7].

The overall body’s response to biomaterials is controlled by factors that determine whether or not the biomaterial is accepted in the body [8]. Since the interaction between the cells or tissues and the biomaterials occurs at their interfaces, the surface properties of the implant are of paramount importance [9].

In recent years, research on methods to improve the properties of Mg alloys is rapidly increasing in order to utilize them as temporary orthopedic implants. Surface engineering has been suggested as an effective tool to control the degradation of Mg alloys and, therefore, make them more effective [10].

Magnesium implants are surface engineered with the aim of improving corrosion resistance, surface adhesion, biocompatibility, stability, uniformity, biomineralization, bone biomaterial fixation, and failure prevention [11, 12]. Application of protective and biocompatible organic coatings is an effective technique applied on Mg alloys [13]. The coatings are applied with different methods such as dip coating [14], spin coating [15], electrophoretic deposition [16, 17] and electrospinning [18].

Electrospinning is a versatile technique that produces continues nanofibers via an electrically charged jet of polymeric solution [19]. Recently, this technique has been used to create biocompatible polymer-based coatings such as collagen [20], PCL [21], PCL/ZnO [22], PCL/Hydroxyapatite [23], ciprofloxacin loaded gelatin [24] for biodegradable Mg alloys. The polymer-based nanofibrous coatings have a large surface to volume ratio, high porosity, and architecture similar to that of extracellular matrix (ECM), which enhances cell adhesion and biocompatibility and also controls corrosion rate of implants in body fluids [25–27]. The important parameters in electrospinning are voltage, feed rate and work distance that affect nanofiber’s structure and nanofibrous coating properties [28].

Chitosan (CS) as a natural polymer is widely used in biomedical applications such as tissue engineering, scaffolds, and bio-coatings due to its biocompatibility and biodegradability as well as its favorable mechanical properties [29–32]. Höhlinger et al. [33] applied CS-bioactive glass coating on a modified surface of WE43 Mg alloy. In another study, Rahimi et al. [34] created nanofibrous coating containing CS and mineralized bone allograft (MBA) nanoparticles on Mg alloy by electrospinning in order to enhance corrosion and biocompatibility behavior in a physiological environment.

Many researchers have reported the application carbon nanotubes (CNTs) to polymeric coatings. Farrokhi-rad et al. [35] showed that the incorporation of carbon nanotubes to a coating enhanced the surface properties. Adding CNTs as a second phase to CS improves its mechanical properties (e.g., Young’s modulus and tensile strength) and electrical conductivity [36, 37]. CNTs also simulate the dimensions of the proteins that make up the native tissue and increase cell adhesion and growth [38]. A recent study aimed to improve the antibacterial properties of a Mg-Zn-Ca alloy by coating with chitosan-based nanofibers with incorporated silver sulfadiazine (AgSD) and multiwall carbon nanotubes (MWCNTs) [39].

Coatings should have proper adhesion to the substrates for improved corrosion resistance and biocompatibility of Mg alloys with body fluids. For this purpose, various surface pre-treatments are performed on the surface of the alloys. Anodizing converts the metallic surface into an anodic oxide layer that affects corrosion resistance and wear properties of the alloy [40]. Anodizing before applying the coatings increases the surface roughness, resulting in greater adhesion of the coating to the substrate [41].

In this study, CS and CS-CNT nanofiber coatings were deposited on pre-treated AZ31 samples via electrospinning. In a previous work published elsewhere, the electrospinning parameters for a CS-CNT nanofibrous coating on an anodized-Mg alloy were optimized [42]. In the present work, the optimized parameters were used for the deposition of the coatings followed by the evaluation of their effects on the corrosion resistance and biocompatibility of the AZ31 Mg alloy.

The structures of the coatings were studied by Field Emission Scanning Electron Microscopy (FE-SEM). The sizes of the nanofibers were calculated by ImageJ software. The elemental compositions of the samples were characterized by Energy Dispersive X-ray Spectroscopy (EDS). Chemical properties of the coatings were analyzed by Fourier Transform Infrared Spectroscopy (FT-IR). Moreover, Raman spectra of the nanofibers were acquired to confirm the presence of CNTs in the fibers. Coating adhesion tests, contact angle tests (wettability), electrochemical corrosion tests, and osteoblast cell culture were also performed to investigate the influence of nanofibrous coatings and anodizing pre-treatment on corrosion resistance and biocompatibility of AZ31 Mg alloys.

## Materials and methods

### Materials

AZ31 magnesium alloy specimens with dimensions of 2 × 2.5 × 0.3 cm were prepared. The chemical composition of the alloy is given in Table 1. Chitosan (CS) with a degree of deacetylation of 85% and molecular weight of 120 KDa from Sigma-Aldrich was considered for electrospinning deposition. Among the CS solvents, trifluoroacetic acid (TFA, Merck) is more suitable for the electrospinning of CS. TFA forms salts with chitosan’s amino groups resulting in the destruction of the rigid interaction between the CS molecules and improves the electrospinnability of the solution. Moreover, the high volatility of TFA helps to speed up evaporation of the electrified jet of CS-TFA solution. To improve the homogeneity of the electrospun CS fibers, dichloromethane (DCM, Merck) was added to the CS-TFA solution (TFA/DCM solvent at 70/30 ratio). Also, to generate a composite coating, multi-wall carbon nanotubes (MWCNTs) (diameter: 50–80 nm, purity = more than 95%, US nano) were added to the solution. Sodium hydroxide (NaOH, Merck) and sodium carbonate (Na_2_CO_3_, Merck) were used for anodizing and stabilization processes, respectively. All chemical solvents and reagents used in this experiment were of laboratory grade.

**Table 1:** Composition of AZ31 magnesium alloy

Element	Al	Mg	Mn	Si	Cu	Fe	Ni	Mg
%wt.	3.0	1.0	0.43	0.01	≤0.01	0.03	≤0.001	Remaining amount

### Anodizing treatment of AZ31 alloy

Mg-based specimens were initially polished with emery paper to 2000 grids and ultrasonically cleaned in acetone, ethanol, and distilled water to remove impurities and grease. The specimens were then placed in 1 M NaOH solution and treated with 3 volts for 10 min by using a stainless steel cathode, resulting in the final anodized samples.

### Electrospinning of CS and CS-CNT nanofibers on AZ31 alloy

The polymeric solution was prepared using a similar method to what has been described previously [43, 44]. Initially, 0.35 g CS was added to a 5 cc solvent of TFA/ DCM (70:30) where it was left to dissolve using a magnetic stirrer for 24 h. To prepare a mixture containing CNT, first, CS was added to 4 cc of the solvent until it was completely dissolved. Next, CNT (1 wt. % of the amount of CS) was also dispersed in 1 cc of the solvent using a 50 W Sonicator for 10 min. A uniform mixture was obtained by adding the dispersed CNT to the CS solution while stirring.

In the next step, the prepared mixture was transferred to a 5-cc syringe. The electrospinning process was carried out under the following conditions: voltage = 20 kV, tip-to-collector distance = 15 cm, and feed rate = 0.5 ml/h. These parameters were optimized to obtain defect-free nanofibers with uniform structure [42].

As a result of the procedures explained, 5 types of samples were obtained. The samples were coded as follows: (a) bare Mg alloy: Mg, (b) Anodized Mg alloy: Mg-A, (c) CS-coated Mg alloy: Mg-C, (d) CS-coated anodized Mg alloy: (Mg-AC), and (e) CS-CNT-coated anodized Mg alloy: (Mg-ACC).

### Stabilization of nanofiber coatings

CS nanofibers lose their fibrous structure when they are exposed to Phosphate- buffered saline (PBS) solution or even 70% ethanol during sterilization [45]. This is because during the dissolution of chitosan in TFA, CS Trifluoroacetate salts (-NH_3_^+^CF_3_COO^-^) are formed between CS chains and TFA molecules that are soluble in aqueous media [46]. Therefore, this problem should be solved before using CS fibers in applications in contact with aqueous media [45]. For this purpose, after 75 min of electrospinning, neutralization of the salts was performed in 5 M super saturated sodium carbonate aqueous solution for 10 min. This duration was decided as a result of the optimization in a previous published work where stabilized nanofibers were obtained with the minimal effect on the morphology of the nanofibers [42].

### Surface characterization

Field emission scanning electron microscopy (FESEM, FEI NOVA NANOSEM 450) was used to observe the morphology of the coated samples. Before FESEM characterization, samples were gold sputter-coated. The average diameter and diameter distribution of nanofibers were measured using ImageJ software by examining 50 fibers in each sample. FESEM was also used to determine the effect of the stabilization process on nanofibers structure. The elemental compositions of the specimens were characterized by Energy Dispersive X-ray Spectroscopy (EDS, CAMSCAN MV2300). Fourier transform infrared (FTIR) spectra were recorded from 4000 to 600 cm^−1^ by using a FTIR Spectrometer (Avatar Nicolet 360). Moreover, the Raman spectra of nanofibers were recorded in a 1000–4200 cm^−1^ range with a Nd: YAG laser source (λ = 532 nm and 0.7 Mw power, TEKSAN N1-541 instrument). To measure the wettability, distilled water droplets were placed on the surface of the samples and the contact angles were measured after 10 s using a contact angle meter (Goniometer, Jikan, CAG-10). The test was performed three times per sample and the values were averaged.

### Electrochemical corrosion test

Corrosion behavior of the samples was analyzed through potentiodynamic polarization and electrochemical impedance spectroscopy (EIS) tests using an EG&G potentiostat-galvanostat equipment (model 273 A). The tests were performed in a simulated body fluid (SBF) solution at 37 °C and pH = 7.4. The total surface area of each sample exposed to the solution was 1 cm^2^. Before the start of the tests, the samples were immersed in the solution for 30 min and a steady state of the open-circuit potential (OCP) was achieved. These tests were conducted using a 3-electrode system including a working electrode (prepared sample), a saturated calomel reference electrode (SCE), and a platinum auxiliary electrode.

In the potentiodynamic polarization test, potential in the range −250 to +250 mV (compared to OCP) was scanned at a rate of 1 mV/s.

The EIS tests were conducted at a frequency range of 100 KHz to 10 mHz with a sinusoidal potential amplitude of 5 mV. All the data obtained from the EIS tests were analyzed using Zview software.

### Coating adhesion test

Effect of anodizing pre-treatment on nanofibrous coating adhesion (to the substrate) was studied using a tape test (according to ASTM D3359–09 standard).

### Immersion test

As mentioned in Section 1, when Mg is placed in an aqueous medium, it gets corroded and Magnesium hydroxide and H_2_ gas are produced as a result.

1 $$Mg + 2H_2O \to Mg^{2 + } + 2OH^ - + H2 \uparrow$$

The amount of the released H_2_ gas is proportional to the number of moles of dissolved Mg. A H_2_ gas evolution test was carried out based on the setup described previously [47]. Each sample was immersed in SBF solution at 37 °C and the released H_2_ gas was measured by collecting it in an inverted measuring cylinder above the specimens. The total surface area exposed to the solution was 1 cm^2^. The immersion test was performed for 72 h. Afterwards, each sample was dried and its surface was studied by SEM and EDS to investigate the effect of the coatings on biomineralization of AZ31 alloy.

### In vitro cytocompatibility assay

The cytocompatibility of the substrates was tested in vitro by measuring the viability of MG63 osteoblasts (Pastor institutes) cultured on the substrates using an MTT assay. The cells were first cultured using Dulbecco’s modified eagle medium (DMEM, containing 10% FBS serum and 1% penicillin/streptomycin). Then, a substrate was placed in each well of 6-well culture plates and osteoblasts were seeded on each sample at a density of 4 × 104 cell/well and stored in a humidified incubator at 37 °C with 5% CO_2_ for 24 or 72 h. An identical volume of cells at the same concentration was seeded into a well without a substrate as a negative control. Then, the culture medium was removed and 1 ml of DMEM containing 10% MTT solution was added to each well. The cells were then incubated for 4 h to form MTT formazan crystals. The MTT solution was replaced by 100 µL of dimethyl sulfoxide solvent (DMSO) to dissolve the crystals. After 15 min, the solution of dissolved formazan crystals was moved to 96-well cell culture plates, and the optical density at the wavelength of 570 nm was measured by a spectrophotometer. For higher reliability, this procedure was repeated 3 times and the average OD was calculated. The cell viability of the samples is calculated using Eq. 2:

2 $$Viability = \left( {OD_S/OD_R} \right) \times 100$$

OD_S_ is the optical density of the specimens and OD_R_ is the optical density of the reference sample.

### Cell adhesion

To evaluate the osteoblast cell adhesion, specimens were first exposed to UV light for 45 min for sterilizing. Afterwards, the cells were cultured on the specimens (4 × 10^4^ cell/cm^2^). After 72 h, the specimens were then removed from the culture medium and were fixed in 4% glutaraldehyde solution for 15 min at 4 °C. Next, the samples were immersed in ethanol (50, 60, 70, 80, 90, and 100% for 10 min of immersion for each). After drying, the surfaces of the specimens were studied using the FESEM.

## Results and discussion

### Surface morphology

**Fig. 1:**
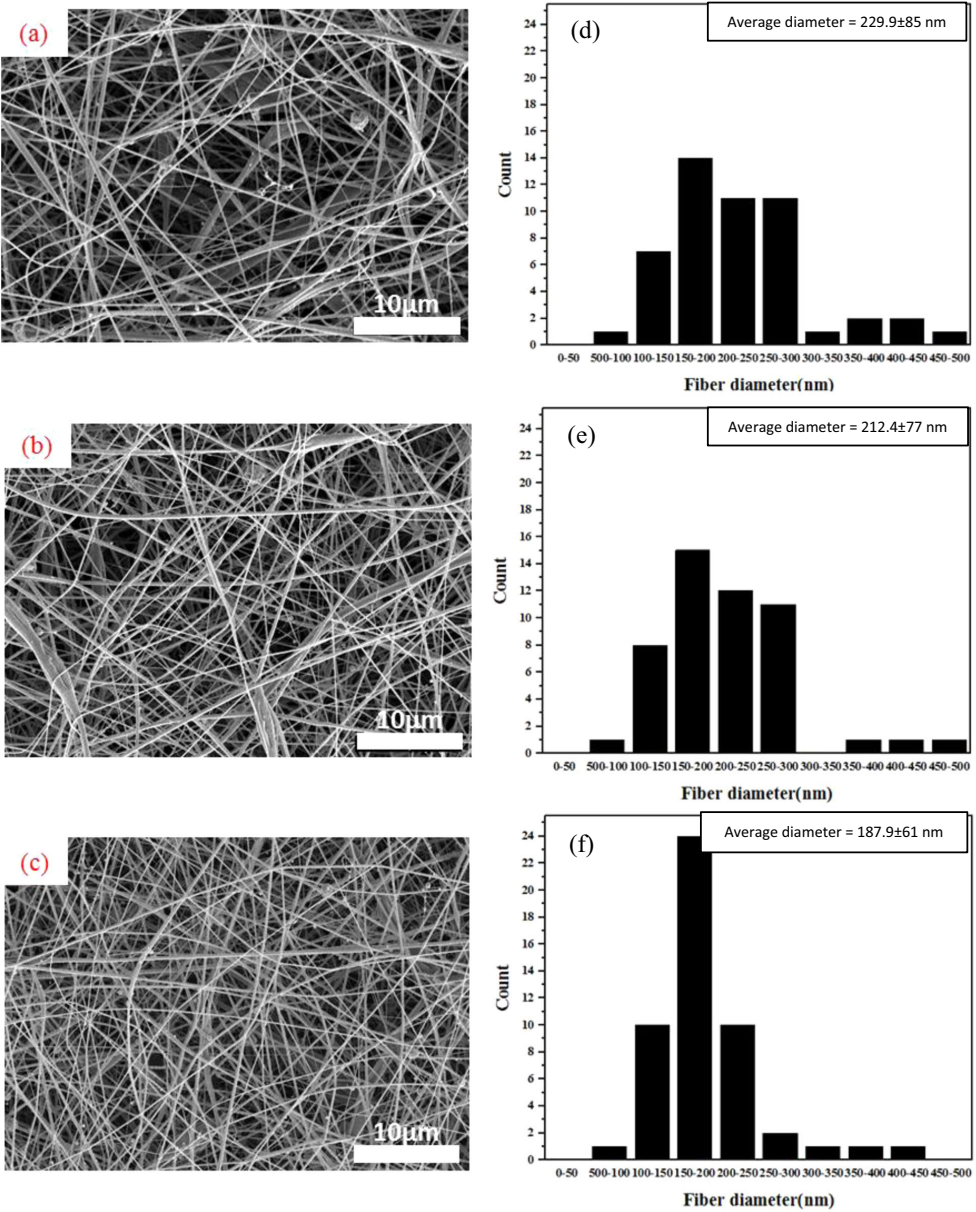
FESEM images of nanofibers; (**a**, **d**) Mg-C, (**b**, **e**) Mg-AC and (**c**, **f**) Mg-ACC.

CS and CS-CNT nanofibers were deposited on pre-treated Mg alloy under the same conditions as explained in Section 2.3. Surface morphology of Mg-C, Mg-AC, and Mg- ACC samples are shown in Fig. 1(a), (b), and (c) respectively, which demonstrate the randomly aligned nanofibrous coatings with interconnected porous structures. Fig. 1(d), (e), and (f) show the fiber diameter histograms corresponding to the samples, including average diameter and standard deviation. Comparison between Fig. 1(b) and (c) and their nanofiber diameters (Fig. 1(e) and (f)) shows that addition of CNT to CS nanofibers resulted in a reduction of the average diameter from 212.4 ± 77 nm to 187.9 ± 61 nm. This is due to the fact that CNT enhances the electrical conductivity of the electrospinning solution, resulting in a higher charge density on the surface of the jet and larger elongation forces during the electrospinning process [48, 49]. Also, compared to Mg-C and Mg-AC, the nanofibers coated on Mg-ACC are more uniform in size and higher in density. The average diameter of the CS nanofibers on the Mg-C sample was 229.9 ± 85 nm (Fig. 1(d)).

**Fig. 2:**
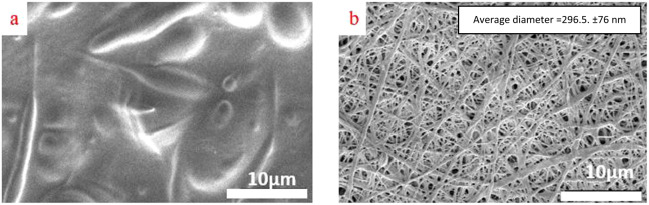
FESEM images of (**a**) non-stabilized specimen after contact with water and (**b**) stabilized specimen in 5 M Na_2_CO_3_ aqueous solution for 10 min

As mentioned in Section 2.4, CS-based nanofibers must be stabilized. So, after electrospinning for 75 min, the coatings were stabilized in 5 M Na_2_CO_3_ aqueous solution. The effect of stabilizing the nanofibers can be observed in Fig. 2: the non-stabilized nanofibrous coating lost its structure after exposure to water (Fig. 2(a)) while the stabilized (for 10 min) coating preserved its structure (Fig. 2 (b)).

### Chemical investigation

**Fig. 3:**
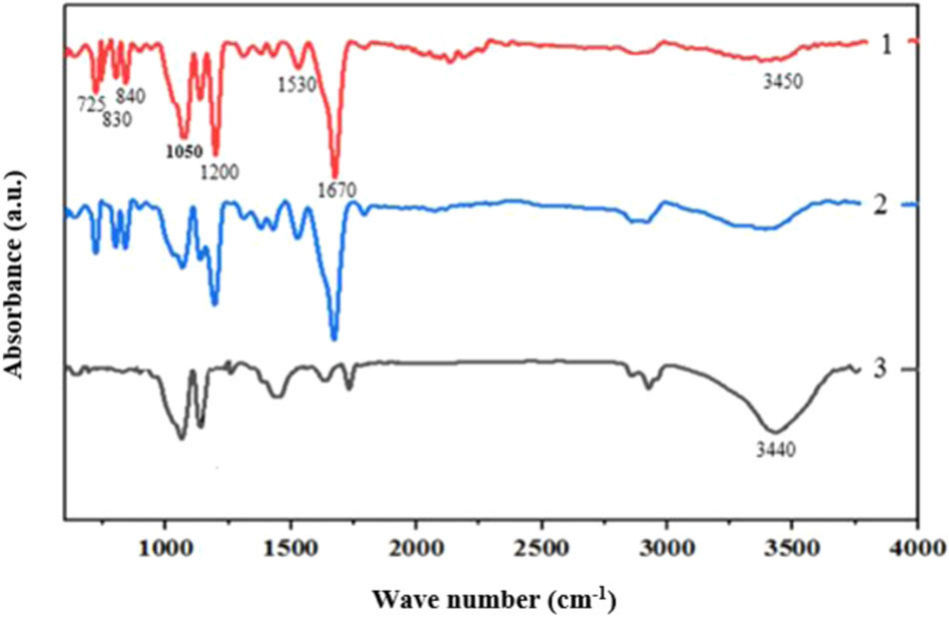
FTIR spectra of nanofibers: (1) CS nanofibers, (2) nanocomposite fibers, and (3) stabilized-CS nanofibers

Figure 3 shows the spectra obtained from FTIR spectroscopy of CS nanofibers, CS-CNT nanofibers, and stabilized CS nanofibers. The addition of CNT slightly changed the spectra peak intensity. The relatively wide peaks at around 3450 cm^−1^ (for all three samples) are associated with the stretching of the primary amine and OH^-^ functional groups. Also, the peaks around 1050 and 1110 cm^−1^ correspond to the saccharide structure of CS. The characteristic peaks of non-stabilized CS and CS-CNT fibers (Fig. 3, spectra (1) and (2)) were observed around 1530 and 1670 cm^−1^, which indicates the stretching of the protonated amine (-NH3^+^) functional group. The presence of a large absorption peak around 1670 cm^−1^, a carboxylate stretching related peak at 1200 cm^−1^, and 3 peaks in the range of 725–840 cm^−1^ (Fig. 3, spectra (1) and (2)) show the presence of TFA in CS fibers as amino salts (-NH_3_^+^CF_3_COO^−^). The amino salts resulted from the dissolution of CS in TFA solvent. In comparison to the non-stabilized samples, the spectrum of the stabilized-CS nanofibers contains a strong peak at about 3440 cm^−1^ which is related to the stretching of the amine functional group (-NH_2_) [45, 50]. In other words, the results confirmed the regeneration of the amine functional groups after the stabilization process.

**Fig. 4:**
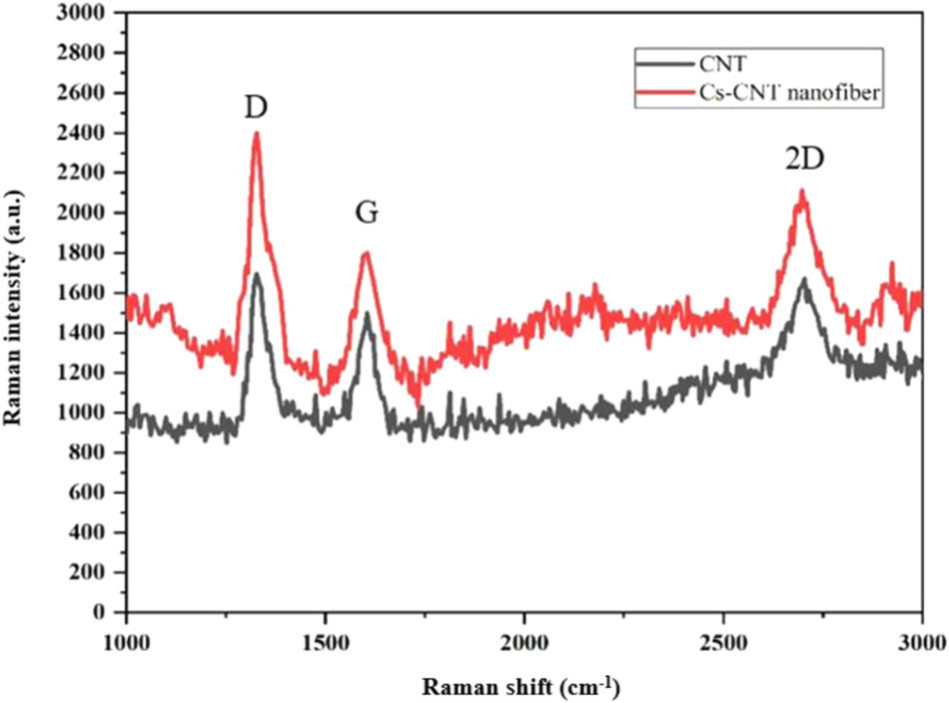
Raman spectra: CNT and CS-CNT nanofibers

The presence of CNTs in CS nanofibers was confirmed by Raman spectroscopy (Fig. 4). It is worth mentioning that the Raman spectra profile of CS-CNT nanofiber coating is highly similar to the spectra of CNTs. In addition, D-band, G-band, and 2D-band peaks are detected at 1332, 1600, and 2680 cm^−1^, respectively. The peaks indicate the presence of CNTs in the nanofibers structure [51, 52]. Moreover, the D-band/G-band peak intensity ratio was increased in the nanofibers compared to that in the CNTs. Also, the stronger D-band peak of the nanofibers (Fig. 4) indicated an increase in structural defects of the CNTs. This is due to the linkages between CNTs and the CS polymer.

### Wettability

**Fig. 5:**
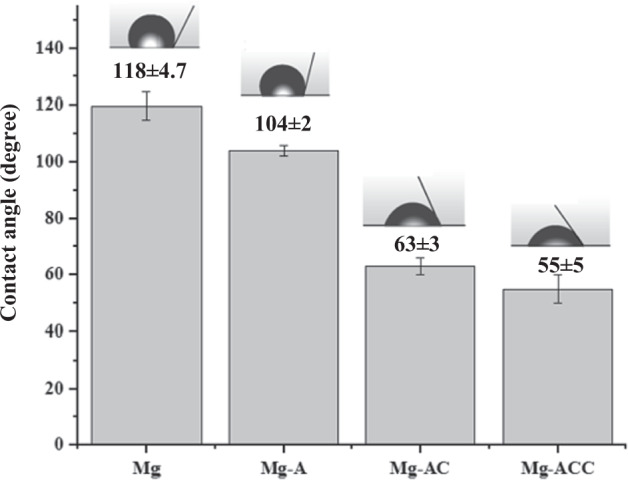
Contact angle measurement results

It is reported that the desired contact angle for orthopedic applications such as bone regeneration is in the hydrophilic range of 35˚to 80˚ [17]. Figure 5 shows the wettability test results of Mg, Mg-A, Mg-AC, and Mg-ACC samples. The schematic form of water droplets on the surface is also shown in Fig. 5.

The contact angle for the Mg sample was 118 ± 4.7° and decreased to 104 ± 2° for the Mg-A sample likely due to its increased surface roughness caused by the pre-treatment process [53]. Another reason for this could be the formation of OH groups on the surface after anodizing. As shown in Fig. 5, the surface of the Mg and Mg-A specimens are hydrophobic (contact angle > 90°). The nanofibrous coated samples exhibited a higher wettability due to the hydrophilic nature of the CS coatings: contact angles for Mg-AC and Mg-ACC samples were 63 ± 3° and 55 ± 5° respectively. A contact angle of 55° (observed for Mg-ACC) has been introduced as an optimal value for bone regeneration applications [19].

### Coating adhesion study

**Fig. 6:**
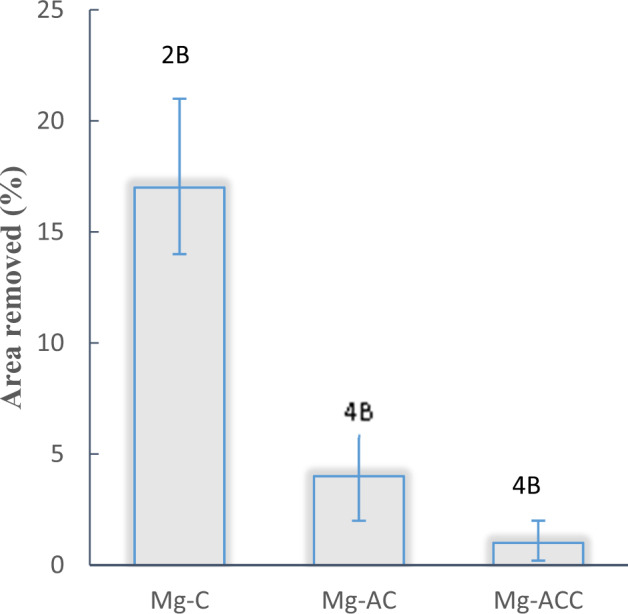
Adhesion performance of the coatings in Mg-C, Mg-AC and Mg-ACC samples

The results of the coating adhesion tests for Mg-C, Mg-AC, and Mg-ACC samples are summarized in Fig. 6. Mg-C had the lowest adhesion (ASTM grade 2B, around 17% of the coating was removed). Anodizing pre-treatment enhances surface roughness and mechanical interactions with the nanofibers, resulting in a higher adhesion. This was confirmed as the detached area in the Mg-AC specimen was reduced to 4% (ASTM grade 4B). Similarly, the removed area for the Mg-ACC specimen was reduced to 1% (ASTM grade 4B).

### Corrosion behavior

**Fig. 7:**
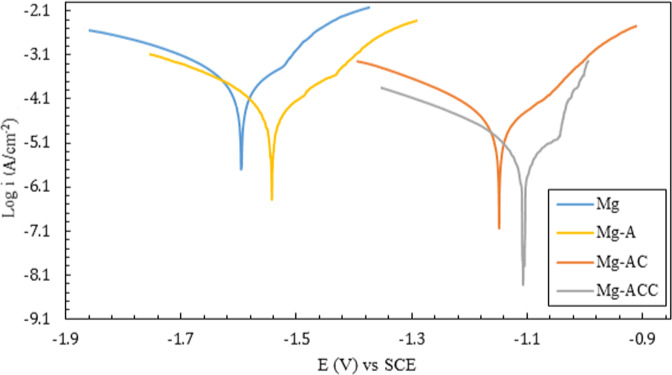
The potentiodynamic polarization curves of different samples

In order to study corrosion behavior of the samples in the SBF solution, they were immersed in the solution and allowed to be equilibrated for 30 min before starting the test, as explained in Section 2.6. The resulting potentiodynamic polarization curves are shown in Fig. 7.

Polarization curves were used to calculate the corrosion rate and potential, the two important parameters to determine the corrosion protection of samples (Table 2). According to previous studies [54–56], higher corrosion potential and lower current density (shown in Table 2) indicate a higher corrosion resistance. According to Fig. 7, current densities for Mg and Mg-A samples were higher than those of the other samples, indicating the higher corrosion rates. The current density of Mg-AC is higher than that of Mg-ACC. Due to the positive effect of the CNTs on the structure of the nanofibers, the Mg-ACC specimen exhibited the highest corrosion resistance. This was confirmed since the anode and cathode branches of this sample are in the lowest position in terms of current density. Additionally, it can be observed from Fig. 7 that the corrosion potential of the nanofibrous coated samples (Mg-AC and Mg-ACC) were changed to the anodic intervals (positively shifted). In terms of thermodynamics, the corrosion resistance for the nanofibrous coated samples increased [57].

**Table 2:** Data obtained from polarization curves

Sample	i_Corr_ (A.cm^−2^)	E_Corr_ (V)	Corrosion rate (mpy)
Mg	2.5 × 10^−4^	−1.596	262
Mg-A	8 × 10^−5^	−1.545	84
Mg-AC	1.6 × 10^−5^	−1.148	17
Mg-ACC	2.5 × 10^−6^	−1.107	2.6

According to Table 2, it is clear that the corrosion current density (and the resulting corrosion rate) of the Mg-ACC sample is about 2 orders of magnitude lower than that of the Mg sample. This is due to a more homogeneous and denser coating of the Mg-ACC specimen. According to Table 2, the corrosion rate of the pre-treated Mg sample (Mg-A) is 84 mpy, which is lower than that of the Mg sample (262 mpy), demonstrating the benefit of the anodizing pre-treatment. Moreover, the potentials of the coated samples have positively shifted: from −1.596 V for the Mg sample to −1.148 and −1.107 V for the Mg-AC and Mg-ACC samples, respectively. Improvement in corrosion resistance of Mg alloy by coating is also confirmed by the results obtained in previous studies [58, 59].

**Fig. 8:**
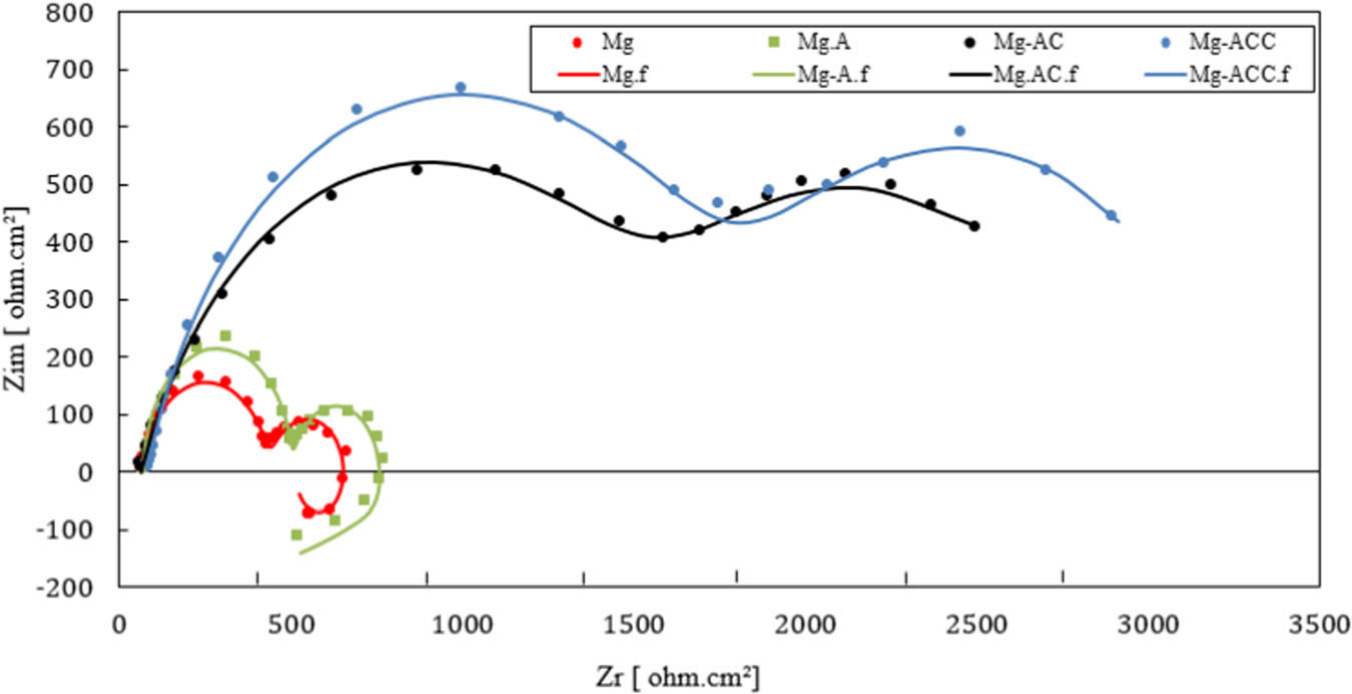
Nyquist plots of Mg, Mg-A, Mg-AC, Mg-ACC samples. The letter f refers to the fitted results

In this section, the results of the EIS test are discussed. The tests were performed for 4 specimens as explained in Section 2.6. Figure 8 shows the Nyquist plot resulting from the EIS test. For the Mg and Mg-A samples, the plot includes two capacitive loops at high frequencies and an inductive loop at low frequencies. The inductive loops indicate the penetration of the corrosive agents into the substrate (pitting corrosion mechanism in Mg alloy) [60, 61]. Mg-AC and Mg-ACC samples did not exhibit an inductive behavior, which indicates the protective nature of the nanofibrous coatings against corrosion (in the SBF solution). As shown in Fig. 8, Mg and Mg-A samples have smaller loop diameters than Mg-AC and Mg-ACC samples. This shows a higher corrosion resistance as a result of CS coating (Mg-AC and Mg-ACC samples).

**Fig. 9:**
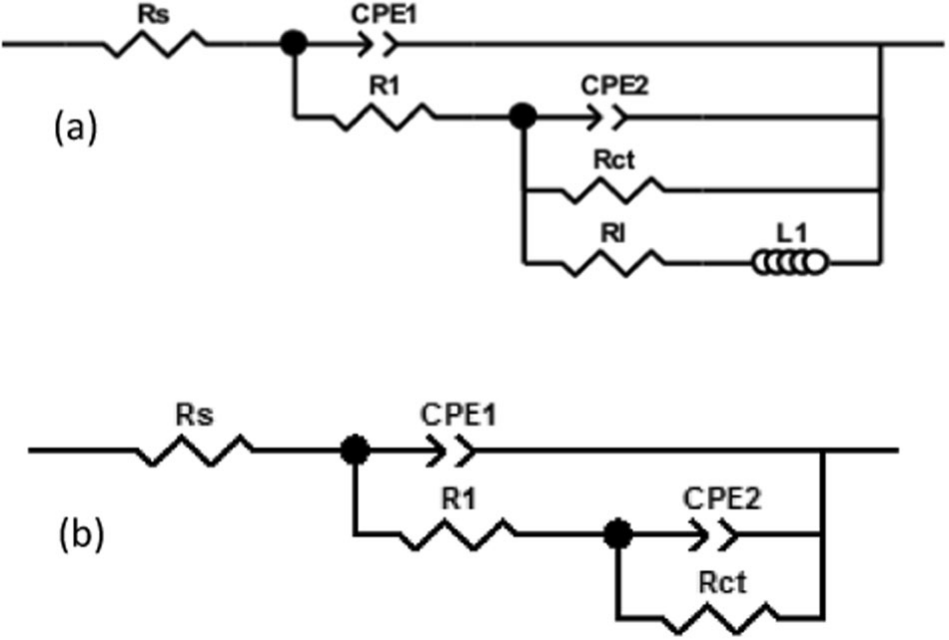
Equivalent circuits; (**a**) for Mg and Mg-A, (**b**) for Mg-AC and Mg-ACC

The equivalent electrical circuits of the samples are presented in Fig. 9. The two capacitive loops (for all of the samples) are shown by the two constant phase elements (CPE_1_ and CPE_2_, capacitor equivalent) (Fig. 9(a) and (b)). The inductive loops presented in the Mg and Mg-A samples are shown by an inductor (L_1_, Fig. 9(a)). The reason for using constant phase elements (instead of capacitors) is the double layer surface roughness. R_s_ and R_ct_ represent the solution and the charge transfer resistances, respectively. R_L_ represents the induction resistance (Fig. 9(a)).

The numerical values of the equivalent circuit elements (Fig. 9) are presented in Table 3. These values are calculated by curve fitting on the EIS test data points (Fig. 8). The fitting and calculations are carried out using Zview software.

**Table 3:** Fitting results of EIS data

Sample	Mg	Mg-A	Mg-AC	Mg-ACC
CPE_1_(Ω^−1^.s^−1^.cm^−2^)	4.68 × 10^−5^	2 × 10^−5^	9.98 × 10^−6^	3.54 × 10^−6^
CPE_2_(Ω^−1^.s^−1^.cm^−2^)	0.0014	0.0013	0.0011	0.0004
n_1_	0.89	0.99	0.74	0.80
n_2_	0.85	0.90	0.75	0.80
R_1_(Ω.cm^2^)	375.6	435	1597	1781
R_s_(Ω.cm^2^)	63.58	67.68	74.32	80
R_ct_ (Ω.cm^2^)	230	262	1219	1408
L(H.cm^2^)	1441	1503	–	–
R_L_(Ω.cm^2^)	107	120	–	–

Higher values of R_ct_ and lower values of CPE indicate a higher corrosion resistance [62]. The positive effect of anodizing pre-treatment can be determined by comparing R_ct_ and CPE for Mg and Mg-A samples: CPE_1_ and CPE_2_ decreased from 4.68 × 10^−5^ and 0.0014 Ω^−1^.s^−1^.cm^−2^ to 2 × 10^−5^ and 0.0013 Ω^−1^.s^−1^.cm^−2^, respectively. R_ct_ increased from 230 to 262 Ω.cm^2^. CS coating further improved the corrosion resistance as CPE_1_ and CPE_2_ decreased to 9.98 × 10^−6^ and 0.0011 Ω^−1^.s^−1^.cm^−2^, respectively and R_ct_ increased to 1219 Ω.cm^2^ for the Mg-AC sample. Finally, it can be observed that Mg-ACC has the highest corrosion resistance since it has the highest value of R_ct_ (1408 Ω.cm^2^) and the lowest value of CPE_1_ (3.54 × 10^−6^ Ω^−1^.s^−1^.cm^−2^) and CPE_2_ (0.0004 Ω^-1^.s^-1^.cm^-2^) compared to the other samples. This shows the superior effect of CS-CNT coating in corrosion resistance (Table 3).

### Immersion test

**Fig. 10:**
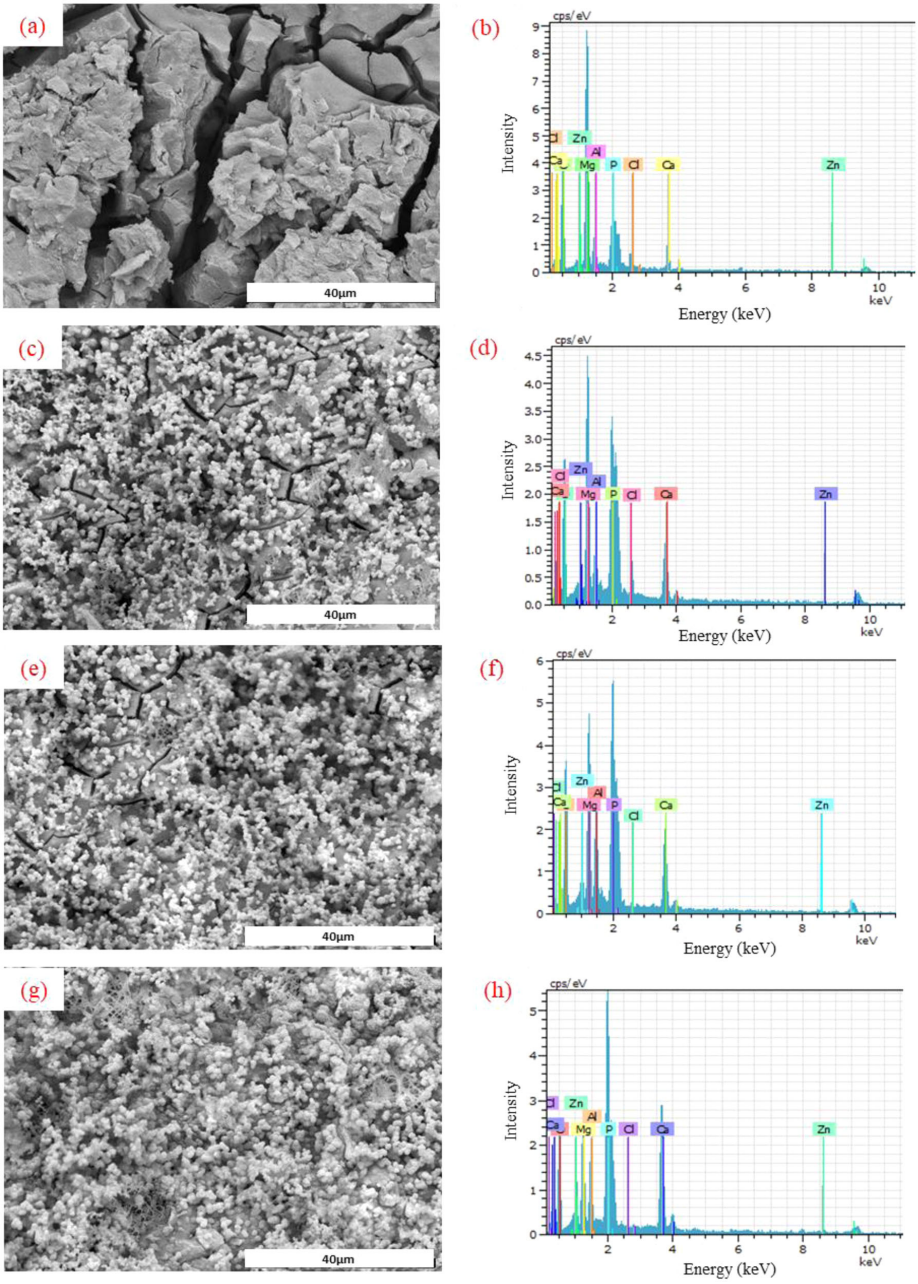
FESEM images and EDS results of the samples after immersion in the SBF solution for 72 h: (**a**) and (**b**) Mg-A; (**c**) and (**d**) Mg-C; (**e**) and **(f**) Mg-AC; (**g**) and (**h**) Mg-ACC

Morphology and chemical composition of the phases formed on the Mg-A, Mg-C, Mg-AC and Mg-ACC samples after immersion in SBF were analyzed using SEM and EDS. SEM images are shown in Fig. 10. Severe degradation and presence of pits on the surface of the Mg-A sample were observed (Fig. 10(a)). Also, surface cracks were found for both the Mg-C and Mg-AC samples (Fig. 10(c, e)). However, the coating remained intact for the Mg-ACC sample after the test (Fig. 10(g)). This is due to higher adhesion of the CS-CNT coating on the anodized substrate (as discussed in Section 3.4).

EDS analysis from particles on the surface of the samples revealed the presence of Mg, Al, Zn, O, Ca, P, and Cl ions which are from the Mg alloy and SBF solution. The presence of these ions suggests the formation of magnesium chloride, magnesium hydroxide, calcium, and magnesium phosphate [63].

The compositions obtained from the EDS of the samples are shown in Fig. 10(b, d, f and h) and Table 4. The surface of the Mg-A sample contains a large amount of Mg and O and a small number of other ions. Compared to Mg-A, other samples showed higher amounts of Ca, P, and O on their surfaces. The Mg-ACC sample had the highest Ca/P ratio, which indicates biomineralization, and the lowest amount of chlorine ion, which indicates pitting corrosion resistance improvement of the Mg alloy. Thus, it can be concluded that the CS-CNT coating has led to a further decrease in the rate of substrate degradation compared to the CS coating, resulting in higher adhesion to the substrate. As a result, the CS-CNT coating is the more suitable substrate for the formation of the calcium phosphate layer during osseointegration.

**Table 4:** Elemental composition of the sample surfaces after immersion for 72 h

Sample	Mg	O	Ca	P	Cl	Al	Zn	Ca/P ratio
Mg-A	29.83	46.09	6.26	8.03	3.9	4.8	1.09	0.78
Mg-C	18.01	45.67	13.2	16.43	2.8	3.3	0.59	0.80
Mg-AC	10.11	46.84	15.58	18.79	2.05	4.6	2.03	0.83
Mg-ACC	6.87	45.89	20.35	22.42	0.28	3.61	0.57	0.91

### Hydrogen evolution test

**Fig. 11:**
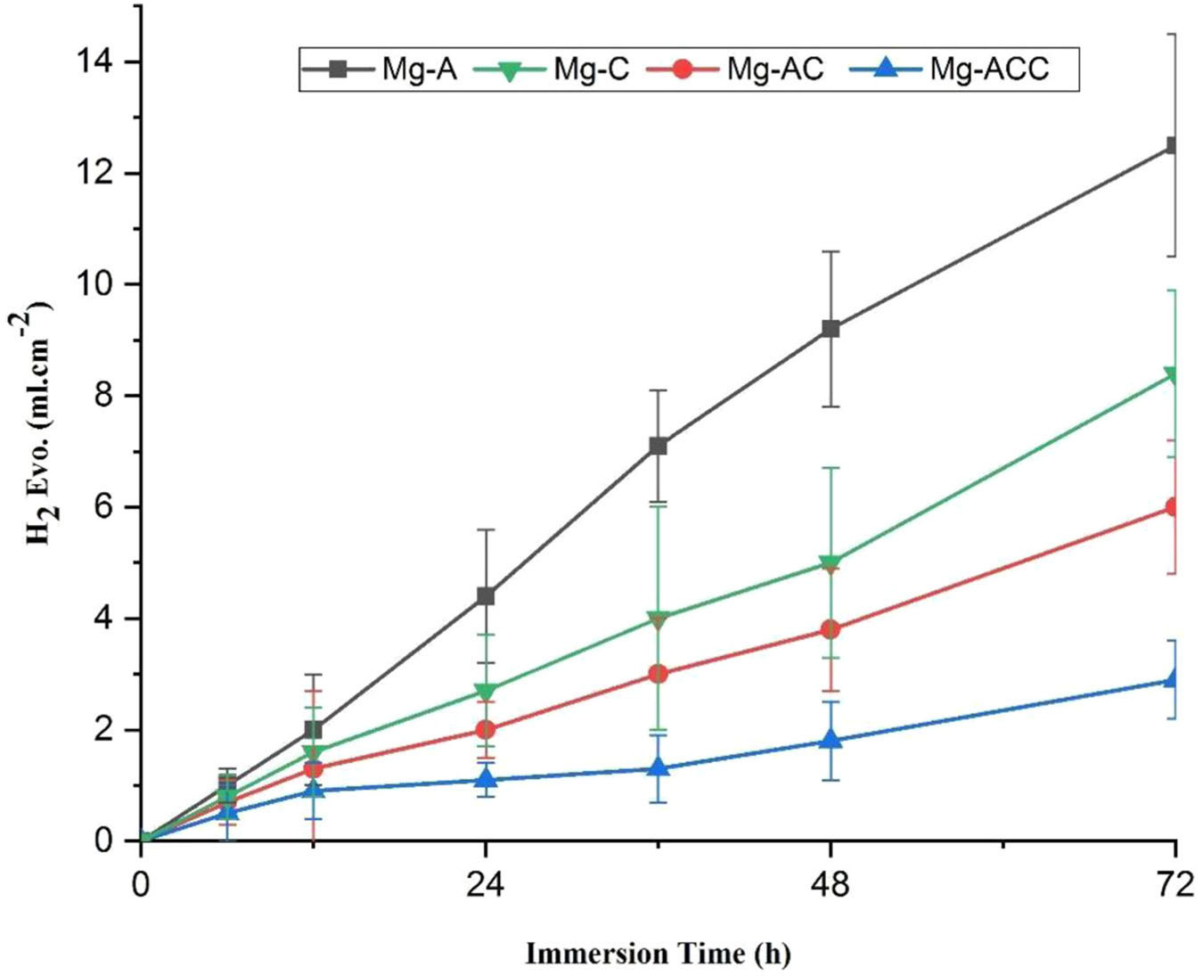
The amount of H_2_ gas released at different times during immersion

A hydrogen evolution test was performed as discussed in Section 2.8. Figure 11 shows the rate of H_2_ gas releasing for the Mg-A, Mg-C, Mg-AC, and Mg-ACC specimens.

The result of the test revealed that the coated samples have a lower rate of H_2_ gas evolution (Mg-A demonstrated the highest rate of release). Reduction in H_2_ gas evolution by creating biocompatible coatings is in agreement with previous studies [9, 38]. The nanofibrous coatings, especially the composite coating, controlled and reduced the H_2_ gas evolution. After three days of immersion, the amount of the released H_2_ gas decreased from 12.5 ml per unit area for Mg-A to 2.9 ml for Mg-ACC. The further reduction of H_2_ evolution for Mg-ACC compared to Mg-AC is due to the more uniform and denser nanofibrous structure and the higher coating adhesion. Furthermore, comparison of Mg-C and Mg-AC results shows that the anodizing process before applying the nanofibrous coatings reduces the amount of H_2_ release. This is due to the increased coating adhesion after the anodizing pre-treatment.

By comparing the amount of chlorine uptake (section 3.6) and H_2_ gas evolution during the degradation of these samples, it was found that there is a relationship between the two (i.e., the sample surface with a higher H_2_ evolution took up more chlorine).

### Cell adhesion and cytotoxicity

**Fig. 12:**
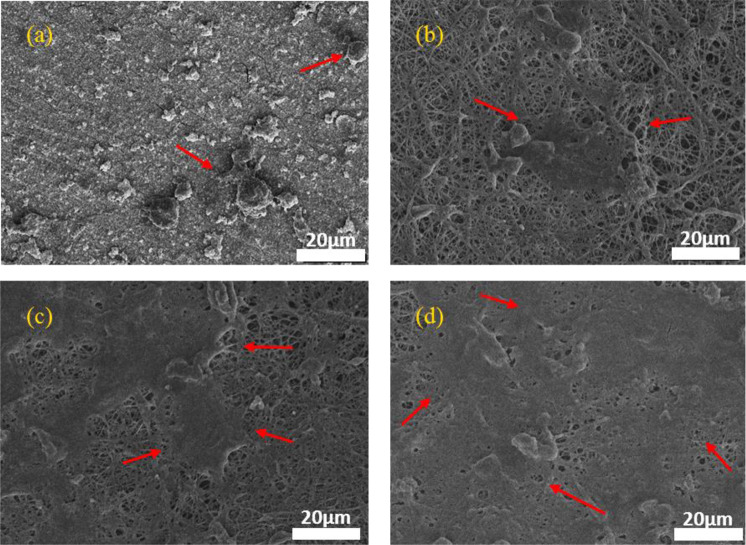
SEM images of samples after cell culture studies for 72 h. **a** Mg-A, (**b**) Mg-C, (**c**) Mg-AC, (**d**) Mg-ACC (The red arrows point to the cells)

Figure 12 shows the surface of the specimens after cell culture studies for 72 h. Compared to the Mg-A sample with individual cells on the surface, mass adhesion of cells is observed on the surface of the coated specimens (due to the higher hydrophilicity and biocompatibility of chitosan-based nanofibrous coated samples). Furthermore, the highest cell adhesion observed on the Mg-ACC specimen (the cells covered almost the entire surface of the Mg-ACC specimen).

**Fig. 13:**
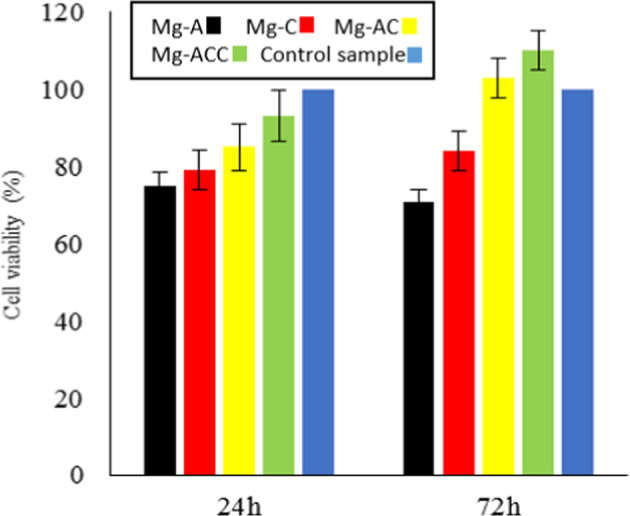
The cell viability for the samples. The viability expressed as a percentage of control

MTT test (see Section 2.9) results after 24 and 72 h are shown in Fig. 13. According to the IOS-10933-5 standard, if the cell viability is above 70%, the sample is non-toxic and biocompatible. Therefore, all of the specimens were determined to be non-toxic. As shown in Fig. 13, cell viabilities for Mg-AC and Mg-ACC specimens are higher than those for Mg-A and Mg-C specimens (especially after 72 h). After the third day, the cell viabilities reached 103 and 110% for Mg-AC and Mg-ACC, respectively, which are about 40 percentage points greater than that of Mg-A. The positive effect of pre-anodizing on improving the biocompatibility of the samples is also observed in Fig. 13. As mentioned in 1, biocompatibility and non-toxicity of the samples depends on various factors such as the amount of toxins released, wettability of the surfaces, coating adhesion to the substrate, and corrosion behavior of the specimen in a physiological environment. Considering the results (Figs. 12 and 13), it can be safely assumed that by applying the nanofibrous composite coating on anodized-AZ31, a suitable substrate was created for cell growth and proliferation.

## Conclusion

In this study, a CS-CNT nanocomposite coating was deposited on AZ31 magnesium alloy via electrospinning. Effects of the pre-treatment and electrospun coatings on corrosion behavior and biocompatibility of the AZ31 alloy were investigated. Anodizing pre-treatment enhanced adhesion of the coating to the substrate and the Mg-ACC specimen gained 4B ASTM grade. The nanofibrous coatings increased corrosion resistance of the Mg alloy in the SBF solution. After immersion in SBF for 72 h, the amount of released H_2_ gas was 12.5 ml per unit area for Mg-A and 2.9 ml for Mg-ACC. Mg-ACC also had the highest Ca/P ratio and the lowest amount of chlorine ion. These nanofibrous coatings exhibited good osteoblast cell adhesion, such that the cells covered almost the entire Mg-ACC area. An MTT assay revealed that, after 72 h, cell viability reached 103 and 110% for Mg-AC and Mg-ACC, respectively, in comparison to the control sample.

## References

[1] Kamrani S, Fleck C (2019). Biodegradable magnesium alloys as temporary orthopaedic implants: a review. Biometals.

[2] Brar HS, Platt MO, Sarntinoranont M, Martin PI, Manuel MV (2009). Magnesium as a biodegradable and bioabsorbable material for medical implants. Jom.

[3] Staiger MP, Pietak AM, Huadmai J, Dias G (2006). Magnesium and its alloys as orthopedic biomaterials: a review. Biomaterials.

[4] Alvarez-Lopez M (2010). Corrosion behaviour of AZ31 magnesium alloy with different grain sizes in simulated biological fluids. Acta biomaterialia.

[5] Song G (2007). Control of biodegradation of biocompatable magnesium alloys. Corros Sci.

[6] Y. Xin, K. Huo, H. Tao, G. Tang, and P. K. Chu, Influence of aggressive ions on the degradation behavior of biomedical magnesium alloy in physiological environment, Acta Biomaterialia. 2008;4:1–15. 10.1016/j.actbio.2008.05.014 18571486

[7] Chakraborty Banerjee P, Al-Saadi S, Choudhary L, Harandi SE, Singh R (2019). Magnesium implants: prospects and challenges. Materials.

[8] Morais JM, Papadimitrakopoulos F, Burgess DJ (2010). Biomaterials/tissue interactions: possible solutions to overcome foreign body response, (in eng). AAPS J.

[9] Wu G, Ibrahim JM, Chu PK (2013). Surface design of biodegradable magnesium alloys—a review. Surf Coat Technol.

[10] Wu G, Li P, Feng H, Zhang X, Chu PK (2015). Engineering and functionalization of biomaterials via surface modification. J Mater Chem B.

[11] Agarwal S, Curtin J, Duffy B, Jaiswal S (2016). Biodegradable magnesium alloys for orthopaedic applications: A review on corrosion, biocompatibility and surface modifications. Mater Sci Eng: C.

[12] Su Y (2016). Improvement of the biodegradation property and biomineralization ability of magnesium–hydroxyapatite composites with dicalcium phosphate dihydrate and hydroxyapatite coatings. ACS Biomater Sci Eng.

[13] Wong HM (2010). A biodegradable polymer-based coating to control the performance of magnesium alloy orthopaedic implants. Biomaterials.

[14] Ma Y (2021). Improved corrosion protective performance of chitosan coatings reinforced with nano-ZnO on degradable magnesium alloy in simulated body fluid. Appl Phys A.

[15] Barani B, Lakshminarayanan A, Subashini R (2019). Microstructural characteristics of chitosan deposited az91 magnesium alloy. Mater Today: Proc.

[16] Hornberger H, Virtanen S, Boccaccini AR (2012). Biomedical coatings on magnesium alloys–a review. Acta biomaterialia.

[17] Moayyeri M, et al. Synthesis of ZrB2–ZrC–SiC ultra-high-temperature nanocomposites by a sol–gel process.Advances in Applied Ceramics. 2018;117:3:189–195.

[18] Bakhsheshi-Rad HR (2019). Coating biodegradable magnesium alloys with electrospun poly-L-lactic acid-åkermanite-doxycycline nanofibers for enhanced biocompatibility, antibacterial activity, and corrosion resistance. Surf Coat Technol.

[19] Ramakrishna S. *An introduction to electrospinning and nanofibers*. World scientific, 2005.

[20] Park CH, Pant HR, Kim CS. Effect on corrosion behavior of collagen film/fiber coated Az31 magnesium Alloy, Digest J Nanomater Biostruct. 2013;8:1227–1234 .

[21] Rezk AI, Mousa HM, Lee J, Park CH, Kim CS (2019). Composite PCL/HA/simvastatin electrospun nanofiber coating on biodegradable Mg alloy for orthopedic implant application. J Coat Technol Res.

[22] Kim J, Mousa HM, Park CH, Kim CS (2017). Enhanced corrosion resistance and biocompatibility of AZ31 Mg alloy using PCL/ZnO NPs via electrospinning. Appl Surf Sci.

[23] Hanas T, Kumar TS, Perumal G, Doble M, Ramakrishna S (2018). Electrospun PCL/HA coated friction stir processed AZ31/HA composites for degradable implant applications. J Mater Process Technol.

[24] Bakhsheshi-Rad H, Hadisi Z, Hamzah E, Ismail A, Aziz M, Kashefian M (2017). Drug delivery and cytocompatibility of ciprofloxacin loaded gelatin nanofibers-coated Mg alloy. Mater Lett.

[25] Hanas T, Kumar TS, Perumal G, Doble M (2016). Tailoring degradation of AZ31 alloy by surface pre-treatment and electrospun PCL fibrous coating. Mater Sci Eng: C.

[26] Yang S, Lei P, Shan Y, Zhang D (2018). Preparation and characterization of antibacterial electrospun chitosan/poly (vinyl alcohol)/graphene oxide composite nanofibrous membrane. Appl Surf Sci.

[27] Rahimi M, Aghdam RM, Sohi MH, Rezayan AH, Ettelaei M (2021). Enhance corrosion behavior of AZ31 magnesium alloy by tailoring the anodic oxidation time followed by heat treatment in simulated body fluid. Anti-Corros Methods Mater.

[28] Haider A, Haider S, Kang I-K (2018). A comprehensive review summarizing the effect of electrospinning parameters and potential applications of nanofibers in biomedical and biotechnology. Arab J Chem.

[29] Aranaz I (2009). Functional characterization of chitin and chitosan. Curr Chem Biol.

[30] Francis A, Yang Y, Boccaccini A (2019). A new strategy for developing chitosan conversion coating on magnesium substrates for orthopedic implants. Appl Surf Sci.

[31] Rinaudo M (2006). Chitin and chitosan: Properties and applications. Prog Polym Sci.

[32] Roshan S, Mohammadloo HE, Sarabi A, Afshari M. Biocompatible hybrid chitosan/hydroxyapatite coating applied on the AZ31 Mg alloy substrate: in-vitro corrosion, surface and structure studies. Mater Today Commun. 2022:1031–53.

[33] Höhlinger M, Heise S, Wagener V, Boccaccini AR, Virtanen S (2017). Developing surface pre-treatments for electrophoretic deposition of biofunctional chitosan-bioactive glass coatings on a WE43 magnesium alloy. Appl Surf Sci.

[34] Rahimi M, Aghdam RM, Sohi MH, Rezayan AH, Ettelaei M (2021). Improving biocompatibility and corrosion resistance of anodized AZ31 Mg alloy by electrospun chitosan/mineralized bone allograft (MBA) nanocoatings. Surf Coat Technol.

[35] Farrokhi-Rad M, Shahrabi T, Mahmoodi S, Khanmohammadi S (2017). Electrophoretic deposition of hydroxyapatite-chitosan-CNTs nanocomposite coatings. Ceram Int.

[36] Shokrgozar MA, Mottaghitalab F, Mottaghitalab V, Farokhi M (2011). Fabrication of porous chitosan/poly(vinyl alcohol) reinforced single-walled carbon nanotube nanocomposites for neural tissue engineering. J Biomed Nanotechnol.

[37] Amirsardari Z (2014). Preparation and characterization of a novel hetero-nanostructure of zirconium diboride nanoparticle-coated multi-walled carbon nanotubes. RSC Adv.

[38] Zhang J, Wen Z, Zhao M, Li G, Dai C (2016). Effect of the addition CNTs on performance of CaP/chitosan/coating deposited on magnesium alloy by electrophoretic deposition. Mater Sci Eng: C.

[39] Bakhsheshi‐Rad HR, Chen X, Ismail AF, Aziz M, Abdolahi E, Mahmoodiyan F (2019). Improved antibacterial properties of an Mg‐Zn‐Ca alloy coated with chitosan nanofibers incorporating silver sulfadiazine multiwall carbon nanotubes for bone implants. Polym Adv Technol.

[40] Salman SA, Okido M. 8 - Anodization of magnesium (Mg) alloys to improve corrosion resistance, in *Corrosion Prevention of Magnesium Alloys*, G.-L. Song, Ed.: Woodhead Publishing, 2013, 197–31.

[41] Yerokhin AL, Nie X, Leyland A, Matthews A, Dowey SJ (1999). Plasma electrolysis for surface engineering. Surf Coat Technol.

[42] Vahedi S, Mehdinavaz Aghdam R, Rezayan AH, Heydarzadeh Sohi M (2020). Carbon nanotubes reinforced electrospun chitosan nanocomposite coating on anodized AZ31 magnesium alloy. J Ultrafine Graine Nanostruct Mater.

[43] Sencadas V (2012). Determination of the parameters affecting electrospun chitosan fiber size distribution and morphology. Carbohydr Polym.

[44] Mahdieh ZM, Mottaghitalab V, Piri N, Haghi AK (2012). Conductive chitosan/multi walled carbon nanotubes electrospun nanofiber feasibility. Korean J Chem Eng.

[45] Sangsanoh P, Supaphol P (2006). Stability improvement of electrospun chitosan nanofibrous membranes in neutral or weak basic aqueous solutions. Biomacromolecules.

[46] Hasegawa M, Isogai A, Onabe F, Usuda M (1992). Dissolving states of cellulose and chitosan in trifluoroacetic acid,”. J Appl Polym Sci.

[47] Chun-Yan Z, Rong-Chang Z, Cheng-Long L, Jia-Cheng G (2010). Comparison of calcium phosphate coatings on Mg–Al and Mg–Ca alloys and their corrosion behavior in Hank’s solution. Surf Coat Technol.

[48] Mazinani S, Ajji A, Dubois C (2010). Fundamental study of crystallization, orientation, and electrical conductivity of electrospun PET/carbon nanotube nanofibers. J Polym Sci Part B: Polym Phys.

[49] Mazinani S, Ajji A, Dubois C (2009). Morphology, structure and properties of conductive PS/CNT nanocomposite electrospun mat. Polymer.

[50] Saderi N, Rajabi M, Akbari B, Firouzi M, Hassannejad Z (2018). Fabrication and characterization of gold nanoparticle-doped electrospun PCL/chitosan nanofibrous scaffolds for nerve tissue engineering. J Mater Sci: Mater Med.

[51] Ostrovidov S (2014). Myotube formation on gelatin nanofibers – Multi-walled carbon nanotubes hybrid scaffolds. Biomaterials.

[52] Amirsardari,Z, et al. Facile Carbothermal Reduction Synthesis of ZrB2 Nanoparticles: The Effect of Starting Precursors. Materials and Manufacturing Processes.2016;31:134–140 .

[53] Torrisi L, Scolaro C (2017). Nanoparticles improving the wetting ability of biological liquids. J Thermodyn Catal.

[54] Singh IB, Singh M, Das S (2015). A comparative corrosion behavior of Mg, AZ31 and AZ91 alloys in 3.5% NaCl solution. J Magnes Alloy.

[55] Atrens A, Song G-L, Cao F, Shi Z, Bowen PK (2013). Advances in Mg corrosion and research suggestions. J Magnes Alloy.

[56] Gaur S, Singh Raman RK, Khanna AS (2014). In vitro investigation of biodegradable polymeric coating for corrosion resistance of Mg-6Zn-Ca alloy in simulated body fluid. Mater Sci Eng: C.

[57] Tian P, Xu D, Liu X (2016). Mussel-inspired functionalization of PEO/PCL composite coating on a biodegradable AZ31 magnesium alloy. Colloids Surf B: Biointerfaces.

[58] Pompa L, Rahman ZU, Munoz E, Haider W (2015). Surface characterization and cytotoxicity response of biodegradable magnesium alloys. Mater Sci Eng: C.

[59] Asadi H, Suganthan B, Ghalei S, Handa H, Ramasamy RP (2021). A multifunctional polymeric coating incorporating lawsone with corrosion resistance and antibacterial activity for biomedical Mg alloys. Prog Org Coat.

[60] Cui L-Y (2017). Corrosion resistance of a self-healing micro-arc oxidation/polymethyltrimethoxysilane composite coating on magnesium alloy AZ31. Corros Sci.

[61] Lim TS, Ryu HS, Hong S-H (2012). Electrochemical corrosion properties of CeO2-containing coatings on AZ31 magnesium alloys prepared by plasma electrolytic oxidation. Corros Sci.

[62] Song YW, Shan DY, Han EH (2007). Comparative study on corrosion protection properties of electroless Ni-P-ZrO_2_ and Ni-P coatings on AZ91D magnesium alloy. Mater Corros.

[63] Song Y, Shan D, Chen R, Zhang F, Han E-H (2009). Biodegradable behaviors of AZ31 magnesium alloy in simulated body fluid. Mater Sci Eng: C.

